# Types for Complexity of Parallel Computation in Pi-Calculus

**DOI:** 10.1007/978-3-030-72019-3_3

**Published:** 2021-03-23

**Authors:** Patrick Baillot, Alexis Ghyselen

**Affiliations:** Imperial College, London, UK; Univ Lyon, CNRS, ENS de Lyon, Universite Claude-Bernard Lyon 1, LIP, 69342 Lyon Cedex 07, France

**Keywords:** Type Systems, Pi-calculus, Process Calculi, Complexity Analysis, Implicit Computational Complexity, Size Types

## Abstract

Type systems as a technique to analyse or control programs have been extensively studied for functional programming languages. In particular some systems allow to extract from a typing derivation a complexity bound on the program. We explore how to extend such results to parallel complexity in the setting of the pi-calculus, considered as a communication-based model for parallel computation. Two notions of time complexity are given: the total computation time without parallelism (the work) and the computation time under maximal parallelism (the span). We define operational semantics to capture those two notions, and present two type systems from which one can extract a complexity bound on a process. The type systems are inspired both by size types and by input/output types, with additional temporal information about communications.

### 1 Introduction

The problem of certifying time complexity bounds for programs is a challenging question, related to the problem of statically inferring time complexity, and it has been extensively studied in the setting of sequential programming languages. One particular approach to these questions is that of type systems, which offers the advantage of providing an analysis which is formally-grounded, compositional and modular. In the functional framework several rich type systems have been proposed, such that if a program can be assigned a type, then one can extract from the type derivation a complexity bound for its execution on any input (see e.g. [21,25,22,20,6,4]). The type system itself thus provides a complexity certification procedure, and if a type inference algorithm is also provided one obtains a complexity inference procedure. This research area is also related to implicit computational complexity, which aims at providing type systems or static criteria to characterize some complexity classes within a programming language (see e.g. [24,13,33,18,15]), and which have sometimes in a second step inspired a complexity certification or inference procedure.

However, while the topic of complexity certification has been thoroughly investigated for sequential programs both for space and time bounds, there only have been a few contributions in the settings of parallel programs and distributed systems. In these contexts, several notions of cost can be of interest to abstract the computation time. First one can wish to know what is during a program execution the total cumulated computation time on all processors. This is called the *work* of the program. Second, one can wonder if an infinite number of processors were available, what would be the execution time of the program when it is maximally parallelized. This is called the *span* or *depth* of the program.

The paper [23] has addressed the problem of analysing the time complexity of programs written in a parallel first-order functional language. In this language one can spawn computations in parallel and use the resulting values in the body of the program. This allows to express a large bunch of classical parallel algorithms. Their approach is based on amortized complexity and builds on a line of work in the setting of sequential languages to define type systems, which allow to derive bounds on the work and the span of the program. However, the language they are investigating does not allow communication between those computations in parallel. Our goal is to provide an approach to analyse the time complexity of programs written in a rich language for communication-based parallel computation, allowing the representation of several synchronization features. We use for that $\pi$-calculus, a process calculus which provides process creation, channel name creation and name-passing in communication. An alternative approach could be to use a language described with session types, as in [9,10]. We will discuss the expressivity for both languages in Section 4.2.

We want to propose methods that, given a parallel program written in $\pi$-calculus, allow to derive upper bounds on its work and span. Let us mention that these notions are not only of theoretical interest. Some classical results provide upper bounds, expressed by means of the work (w) and span (s), on the evaluation time of a parallel program on a given number $p$ of processors. For instance such a program can be evaluated on a shared-multiprocessor system (SMP) with $p$ processors in time $O(\max(w/p, s))$ (see e.g. [19]).

Our goal in this paper is essentially fundamental and methodological, in the sense that we aim at proposing type systems which are general enough, well-behaved and provide good complexity properties. We do not focus yet at this stage on the design and efficiency of type inference algorithms.

We want to be able to derive complexity bounds which are parametric in the size of inputs, for instance which depend on the length of a list. For that it will be useful to have a language of types that can carry information about sizes, and for this reason we take inspiration from size types [26,6]. So data-types will be annotated with an index which will provide some information on the size of values. Our approach then follows the standard approach to typing in the $\pi$-calculus, namely typing a channel by providing the types of the messages that can be sent or received through it. Actually a second ingredient will be necessary for us, input/output types. In this setting a channel is given a set of capabilities: it can be an input, an output, or have both input/output capabilities.

**Contributions.** We consider a $\pi$-calculus with an explicit `tick` construction; this allows to specify several cost models, instead of only counting the number of reduction steps. Two semantics of this $\pi$-calculus are proposed to define formally the work and the span of a process. We then design two type systems for the $\pi$-calculus, one for the work and one for the span, and establish for both a soundness theorem: if a process is well-typed in the first (resp. second) type system, then its type provides an expression which, for its execution on any input, bounds the work (resp. span). This approach by type system is generic: the soundness proof relies on subject reduction, and it gives a compositional and flexible result that could be adapted to extensions of the base language.

**Discussion.** Note that even though one of the main usages of $\pi$-calculus is to specify and analyse concurrent systems, the present paper does not aim at analysing the complexity of arbitrary $\pi$-calculus concurrent programs. Indeed, some typical examples of concurrent systems like semaphores will simply not be typable in the system for span (see Sect. 4.2), because of linearity conditions. As explained above, our interest here is instead focused on parallel computation expressed in the $\pi$-calculus, which can include some form of cooperative concurrency. We believe the analysis of complexity bounds for concurrent $\pi$-calculus is another challenging question, which we want to address in future work.

A comparison with related works will be done in Sect. 6.

### 2 The Pi-calculus with Semantics for Work and Span

In this work, we consider the $\pi$-calculus as a model of parallelism. The main points of $\pi$-calculus are that processes can be composed in parallel, communication between processes happens with the use of channels, and channel names can be created dynamically.

#### 2.1 Syntax, Congruence and Standard Semantics for $\pi$-Calculus

We present here a classical syntax for the asynchronous $\pi$-calculus. More details about $\pi$-calculus and variants of the syntax can be found in [34]. We define the sets of *variables*, *expressions* and *processes* by the following grammar.

$$v := x, y, z \;\Big|\; a, b, c \qquad e := v \;\Big|\; \mathtt{0} \;\Big|\; \mathtt{s}(e) \;\Big|\; [] \;\Big|\; e :: e'$$

$$P, Q := 0 \;\Big|\; (P \mid Q) \;\Big|\; !a(\tilde{v}).P \;\Big|\; a(\tilde{v}).P \;\Big|\; \overline{a}\langle \tilde{e} \rangle \;\Big|\; (\nu a)P \;\Big|\; \mathtt{tick}.P$$

$$\Big|\; \mathtt{match}(e)\ \{\mathtt{0} \mapsto P;;\ \mathtt{s}(x) \mapsto Q\} \;\Big|\; \mathtt{match}(e)\ \{[] \mapsto P;;\ x :: y \mapsto Q\}$$

Variables $x, y, z$ denote *base type variables*, they represent integers or lists. Variables $a, b, c$ denote *channel names*. The notation $\tilde{v}$ stands for a sequence of variables $v_1, v_2, \ldots, v_k$. In the same way, $\tilde{e}$ is a sequence of expressions. We work up to $\alpha$-renaming, and we write $P[\tilde{v} := \tilde{e}]$ to denote the substitution of the free variables $\tilde{v}$ in $P$ by $\tilde{e}$. For the sake of simplicity, we consider only integers and lists as base types in the following, but the results can be generalized to other algebraic data-types.

Intuitively, $P \mid Q$ stands for the parallel composition of $P$ and $Q$, $a(\tilde{v}).P$ represents an input: it stands for the reception on the channel $a$ of a tuple of values identified by the variables $\tilde{v}$ in the continuation P. The process $!a(\tilde{v}).P$ is a replicated version of $a(\tilde{v}).P$, it behaves like an infinite number of $a(\tilde{v}).P$ in parallel. The process $\overline{a}\langle\tilde{e}\rangle$ represents an output: it sends a sequence of expressions on the channel $a$. A process $(\nu a)P$ dynamically creates a new channel name $a$ and then proceeds as $P$. We also have classical pattern matching on data types, and finally, in $\texttt{tick}.P$, the tick incurs an additional cost of one. This constructor is the source of time complexity in a program. It can represent different cost models and it is more general than only counting the number of reduction steps. For example, by adding a `tick` after each input, we can count the number of communications in a process. By adding it after each replicated input on a channel $a$, we can count the number of calls to $a$. And if we want to count the number of reduction steps, we can add a `tick` after each input and pattern matching.

We can now describe the classical semantics for this calculus. We first define on those processes a congruence relation $\equiv$ : this is the least congruence relation closed under:

$$P \mid 0 \equiv P \qquad P \mid Q \equiv Q \mid P \qquad P \mid (Q \mid R) \equiv (P \mid Q) \mid R$$

$$(\nu a)(\nu b)P \equiv (\nu b)(\nu a)P \qquad (\nu a)(P \mid Q) \equiv (\nu a)P \mid Q \text{ (when } a \text{ is not free in } Q)$$

Note that the last rule can always be applied from right to left by $\alpha$-renaming. Also, one can see that contrary to usual congruence relation for the $\pi$-calculus, we do not consider the rule for replicated input ($!P \equiv\ !P \mid P$) as it will be captured by the semantics, and $\alpha$-conversion is not taken as an explicit rule in the congruence. By associativity, we will often write parallel composition for any number of processes and not only two. Another way to see this congruence relation is that, up to congruence, a process is entirely described by a set of channel names and a multiset of processes. Formally, we can give the following definition.

**Definition 1 (Guarded Processes and Canonical Form).** *A process $G$ is* guarded *if it has one of the following shapes:*

$$G := !a(\tilde{v}).P \;\Big|\; a(\tilde{v}).P \;\Big|\; \overline{a}\langle\tilde{e}\rangle \;\Big|\; \texttt{tick}.P \;\Big|$$

$$\texttt{match}(e)\ \{\texttt{0} \mapsto P;;\ \texttt{s}(x) \mapsto Q\} \;\Big|\; \texttt{match}(e)\ \{[] \mapsto P;;\ x :: y \mapsto Q\}$$

*We say that a process is in* canonical form *if it has the form $(\nu\tilde{a})(G_1 \mid \cdots \mid G_n)$ with $G_1, \ldots, G_n$ guarded processes.*

The properties of this canonical form can be found in the technical report [5], here we only use it to give an intuition of how one could understand a process. Thus, it is enough to consider that for each process $P$, there is a process in canonical form congruent to $P$. Moreover, this canonical form is unique up to the ordering of names and processes, and up to congruence inside guarded processes.

We can now define the usual reduction relation for the $\pi$-calculus, that we denote $P \to Q$. It is defined by the rules given in Figure 1. The rules for integers are not detailed as they can be deduced from the ones for lists. Remark that substitution should be well-defined in order to do some reduction steps: channel names must be substituted by other channel names and base type variables can be substituted by any expression except channel names. However, when we will consider typed processes, this will always yield well-defined substitutions.

$$\frac{}{!a(\tilde{v}).P \mid \overline{a}\langle\tilde{e}\rangle \to !a(\tilde{v}).P \mid P[\tilde{v} := \tilde{e}]} \qquad \frac{}{a(\tilde{v}).P \mid \overline{a}\langle\tilde{e}\rangle \to P[\tilde{v} := \tilde{e}]}$$

$$\frac{}{\mathtt{match}([])\ \{[] \mapsto P;;\ x :: y \mapsto Q\} \to P}$$

$$\frac{}{\mathtt{match}(e :: e')\ \{[] \mapsto P;;\ x :: y \mapsto Q\} \to Q[x, y := e, e']}$$

$$\frac{P \to Q}{P \mid R \to Q \mid R} \qquad \frac{P \to Q}{(\nu a)P \to (\nu a)Q} \qquad \frac{P \equiv P' \qquad P' \to Q' \qquad Q' \equiv Q}{P \to Q}$$

**Fig. 1.** Standard Reduction Rules

For now, this relation cannot reduce a process of the form $\mathtt{tick}.P$. So, we need to introduce a reduction rule for $\mathtt{tick}$. From this semantics, we will define a reduction corresponding to total complexity (*work*). Then, we will define parallel complexity (*span*) by taking an expansion of the standard reduction.

### 2.2 Semantics and Complexity

**Work.** We first describe a semantics for the work, that is to say the total number of ticks during a reduction without parallelism. The time reduction $\to_1$ is defined in Figure 2. Intuitively, this reduction removes exactly one tick at the top-level.

$$\frac{}{\mathtt{tick}.P \to_1 P}$$

$$\frac{P \to_1 P'}{P \mid Q \to_1 P' \mid Q} \qquad \frac{Q \to_1 Q'}{P \mid Q \to_1 P \mid Q'} \qquad \frac{P \to_1 P'}{(\nu a)P \to_1 (\nu a)P'}$$

**Fig. 2.** Simple Tick Reduction Rules

Then from any process $P$, a sequence of reduction steps to $Q$ is just a sequence of one-step reductions with $\to$ or $\to_1$, and the work complexity of this sequence is the number of $\to_1$ steps. In this paper, we always consider the worst-case complexity so the work of a process is defined as the maximal complexity over all such sequences of reduction steps from this process.

Notice that with this semantics for work, adding `tick` in a process does not change its behaviour: we do not create nor erase reduction paths.

**Span.** A more interesting notion of complexity in this calculus is the parallel one. Before presenting the semantics, we present with some simple examples what kind of properties we want for this parallel complexity.

First, we want a parallel complexity that works as if we had an infinite number of processors. So, on the process $\texttt{tick}.0 \mid \texttt{tick}.0 \mid \texttt{tick}.0 \mid \cdots \mid \texttt{tick}.0$ we want the complexity to be 1, whatever the number of `tick` in parallel.

Moreover, reductions with a zero-cost complexity (in our setting, this should mean all reductions except when we reduce a `tick`) should not harm this maximal parallelism. For example $a().\texttt{tick}.0 \mid \overline{a}\langle\rangle \mid \texttt{tick}.0$ should also have complexity one, because intuitively this synchronization between the input and the output can be done independently of the `tick` on the right, and then the `tick` on the left can be reduced in parallel with the `tick` on the right.

Finally, as before for the work, adding a `tick` should not change the behaviour of a process. For instance, consider the process $\texttt{tick}.a().P_0 \mid a().\texttt{tick}.P_1 \mid \overline{a}\langle\rangle$, where $a$ is not used in $P_0$ and $P_1$. This process should have the complexity $max(1 + C_0, 1 + C_1)$, where $C_i$ is the cost of $P_i$. Indeed, there are two possible reductions, either we reduce the tick, and then we synchronize the left input with the output, and continue with $P_0$, or we first do the synchronization with the right input and the output, we then reduces the ticks and finally we continue as $P_1$.

A possible way to define such a parallel complexity would be to take causal complexity [13,12,11], however we believe there is a simpler presentation for our case. In the technical report [5], we prove the equivalence between causal complexity and the notion presented here. The idea has been proposed by Naoki Kobayashi (private communication). It consists in introducing a new construction for processes, $m : P$, where $m$ is an integer. A process using this constructor will be called an *annotated process*. Intuitively, this annotated process has the meaning *$P$ with $m$ ticks before.* We can then enrich the congruence relation $\equiv$ with the following rules:

$$m : (P \mid Q) \equiv (m : P) \mid (m : Q) \qquad m : (\nu a)P \equiv (\nu a)(m : P)$$

$$m : (n : P) \equiv (m + n) : P \qquad 0 : P \equiv P$$

This intuitively means that the ticks can be distributed over parallel composition, name creation can be done before or after ticks without changing the semantics, ticks can be grouped together, and finally zero tick is equivalent to nothing.

With this congruence relation and this new constructor, we can give a new shape to the canonical form presented in Definition 1.

**Definition 2 (Canonical Form for Annotated Processes).** *An annotated process is in canonical form if it has the shape:*

$$(\nu\tilde{a})(n_1 : G_1 \mid \cdots \mid n_m : G_m)$$

*with $G_1, \dots, G_m$ guarded annotated processes.*

Remark that the congruence relation above allows to obtain this canonical form from any annotated processes. With this intuition in mind, we can then define a reduction relation $\Rightarrow_p$ for annotated processes. The rules are given in Figure 3. We do not detail the rules for integers as they are deducible from the ones for lists. Intuitively, this semantics works as the usual semantics for pi-calculus, but when doing a synchronization, we keep the maximal annotation, and ticks are memorized in the annotations.

$$\frac{}{(n : a(\tilde{v}).P) \mid (m : \overline{a}\langle\tilde{e}\rangle) \Rightarrow_p (max(m,n) : P[\tilde{v} := \tilde{e}])} \qquad \frac{}{\texttt{tick}.P \Rightarrow_p 1 : P}$$

$$\frac{}{(n : !a(\tilde{v}).P) \mid (m : \overline{a}\langle\tilde{e}\rangle) \Rightarrow_p (n : !a(\tilde{v}).P) \mid (max(m,n) : P[\tilde{v} := \tilde{e}])}$$

$$\frac{}{\texttt{match}([])\ \{[] \mapsto P;;\ x :: y \mapsto Q\} \Rightarrow_p P}$$

$$\frac{}{\texttt{match}(e :: e')\ \{[] \mapsto P;;\ x :: y \mapsto Q\} \Rightarrow_p Q[x, y := e, e']}$$

$$\frac{P \Rightarrow_p Q}{P \mid R \Rightarrow_p Q \mid R} \qquad \frac{P \Rightarrow_p Q}{(\nu a)P \Rightarrow_p (\nu a)Q} \qquad \frac{P \Rightarrow_p Q}{(n : P) \Rightarrow_p (n : Q)}$$

$$\frac{P \equiv P' \qquad P' \Rightarrow_p Q' \qquad Q' \equiv Q}{P \Rightarrow_p Q}$$

**Fig. 3.** Reduction Rules

We can then define the parallel complexity of an annotated process.

**Definition 3 (Parallel Complexity).** *Let $P$ be an annotated process. We define its* local complexity $\mathcal{C}_\ell(P)$ *by:*

$$\mathcal{C}_\ell(n : P) = n + \mathcal{C}_\ell(P) \qquad \mathcal{C}_\ell(P \mid Q) = \max(\mathcal{C}_\ell(P), \mathcal{C}_\ell(Q))$$

$$\mathcal{C}_\ell((\nu a)P) = \mathcal{C}_\ell(P) \qquad \mathcal{C}_\ell(G) = 0 \textit{ if } G \textit{ is a guarded process}$$

*Equivalently, $\mathcal{C}_\ell(P)$ is the maximal integer that appears in the canonical form of $P$. Then, for an annotated process $P$, its* global parallel complexity *is given by $max\{n \mid P \Rightarrow_p^* Q \wedge \mathcal{C}_\ell(Q) = n\}$ where $\Rightarrow_p^*$ is the reflexive and transitive closure of $\Rightarrow_p$.*

To show that this parallel complexity is well-behaved, we give the following lemma.

**Lemma 1 (Reduction and Local Complexity).** *Let $P, P'$ be annotated processes such that $P \Rightarrow_p P'$. Then, we have $\mathcal{C}_\ell(P') \geq \mathcal{C}_\ell(P)$.*

This lemma is proved by induction. The main point is that guarded processes have a local complexity equal to zero, so doing a reduction will always increase this local complexity. Thus, in order to bound the complexity of an annotated process, we need to reduce it with $\Rightarrow_p$, and then we have to take the maximum local complexity over all normal forms. Moreover, this semantics respects the conditions given in the beginning of this section.

#### 2.3 An Example Process

As an example, we show a way to encode a usual functional program in $\pi$-calculus. In order to do this, we use replicated input to encode functions, and we use a return channel for the output. So, given a channel $f$ representing a function $F$ such that $\overline{f}\langle y, a\rangle$ returns $F(y)$ on the channel $a$, we can write the "map" function in our calculus as described in Figure 4. The main idea for this kind of encoding is to use the dynamic creation of names $\nu$ to create the return channel before calling a function, and then to use this channel to obtain back the result of this call. Note that we chose here as cost model the number of calls to $f$, and we can see the versatility of a `tick` constructor instead of a complexity that relies only on the number of reduction steps.

With this process, on a list of length $n$, the work is $n$. However, as all calls to $f$ could be done in parallel, the span is 1 for any non-empty list as input.

$$
\begin{array}{l}
!\mathrm{map}(x, f, a).\ \mathtt{match}(x)\ \{\\
\quad [\,] \mapsto\ \overline{a}\langle x\rangle\ \ ;;\\
\quad y :: x_1 \mapsto\ (\nu b)(\nu c)(\mathtt{tick}.\overline{f}\langle y, b\rangle\ \mid\ \overline{\mathrm{map}}\langle x_1, f, c\rangle\ \mid\ b(z).c(x_2).\overline{a}\langle z :: x_2\rangle)\\
\}
\end{array}
$$

**Fig. 4.** The Map Function

### 3 Size Types for the Work

We now define a type system to bound the work of a process. The goal is to obtain a soundness result: if a process $P$ is typable then we can derive an integer expression $K$ such that the work of $P$ is bounded by $K$.

#### 3.1 Size Input/Output Types

Our type system relies on the definition of indices to keep track of the size of values in a process. Those indices were for example used in [6] and are greatly inspired by [26]. The main idea of those types in a sequential setting is to control recursive calls by ensuring a decreasing in the sizes.

**Definition 4.** *The set of* indices *for natural numbers is given by the following grammar.*

$$I, J, K := i, j, k \;\Big|\; f(I_1, \ldots, I_n)$$

*The variables $i, j, k$ are called index variables. The set of index variables is denoted $\mathcal{V}$. The symbol $f$ is an element of a given set of function symbols containing addition and multiplication. We also assume that we have the subtraction as a function symbol, with $n - m = 0$ when $m \geq n$. Each function symbol $f$ of arity $\mathtt{ar}(f)$ comes with an interpretation $[\![f]\!] : \mathbb{N}^{\mathtt{ar}(f)} \to \mathbb{N}$.*

Given an index valuation $\rho : \mathcal{V} \to \mathbb{N}$, we extend the interpretation of function symbols to indices, noted $[\![I]\!]_\rho$ in the natural way. In an index $I$, the substitution of the occurrences of $i$ in $I$ by $J$ is denoted $I\{J/i\}$.

**Definition 5 (Constraints on Indices).** *Let $\phi \subset \mathcal{V}$ be a set of index variables. A* constraint *$C$ on $\phi$ is an expression with the shape $I \bowtie J$ where $I$ and $J$ are indices with free variables in $\phi$ and $\bowtie$ denotes a binary relation on integers. Usually, we take $\bowtie \in \{\leq, <, =, \neq\}$. Finite set of constraints are denoted $\Phi$.*

For a set $\phi \subset \mathcal{V}$, we say that a valuation $\rho : \phi \to \mathbb{N}$ *satisfies* a constraint $I \bowtie J$ on $\phi$, noted $\rho \vDash I \bowtie J$ when $[\![I]\!]_\rho \bowtie [\![J]\!]_\rho$ holds. Similarly, $\rho \vDash \Phi$ holds when $\rho \vDash C$ for all $C \in \Phi$. Likewise, we note $\phi; \Phi \vDash C$ when for all valuations $\rho$ on $\phi$ such that $\rho \vDash \Phi$ we have $\rho \vDash C$. Remark that the order $\leq$ in a context $\phi; \Phi$ is not total in general, for example $(i, j); \cdot \nvDash i \leq ij$ and $(i, j); \cdot \nvDash ij \leq i$.

**Definition 6.** *The set of* base types *is given by the following grammar.*

$$\mathcal{B} := \mathsf{Nat}[I, J] \;\Big|\; \mathsf{List}[I, J](\mathcal{B})$$

Intuitively, an integer $n$ of type $\mathsf{Nat}[I, J]$ must be such that $I \leq n \leq J$. Likewise, a list of type $\mathsf{List}[I, J](\mathcal{B})$ must have a length between $I$ and $J$. With those types comes a notion of subtyping, in order to have some flexibility on bounds. This is described by the rules of Figure 5. In a subtyping judgement $\phi; \Phi \vdash T \sqsubseteq T'$ the free index variables of $T$, $T'$, $\Phi$ should be included in $\phi$.

$$\frac{\phi; \Phi \vDash I' \leq I \qquad \phi; \Phi \vDash J \leq J'}{\phi; \Phi \vdash \mathsf{Nat}[I, J] \sqsubseteq \mathsf{Nat}[I', J']}$$

$$\frac{\phi; \Phi \vDash I' \leq I \qquad \phi; \Phi \vDash J \leq J' \qquad \phi; \Phi \vdash \mathcal{B} \sqsubseteq \mathcal{B}'}{\phi; \Phi \vdash \mathsf{List}[I, J](\mathcal{B}) \sqsubseteq \mathsf{List}[I', J'](\mathcal{B}')}$$

**Fig. 5.** Subtyping Rules for Base Size Types

Then, after base types, we have to give a type to channel names in a process. As we want to generalize subtyping for channel types, we will use input/output types [34]. Intuitively, in such a type, in addition to the types that can be sent and received for a channel, a channel is given a set of capabilities: either it is both an input and output channel, or it has only one of those capabilities. This is useful in order to use subtyping, as an input channel and an output channel do not behave in the same way with regards to subtyping. Indeed, an input/output channel is invariant for subtyping, an input channel is covariant and an output channel is contravariant. Unlike in usual input/output types, in this work we also distinguish two kinds of channels : the *simple channels* (that we will often call channels), and replicated channels (called *servers*).

**Definition 7.** *The set of types is given by the following grammar.*

$$T := \mathcal{B} \;\big|\; \mathsf{ch}(\tilde{T}) \;\big|\; \mathsf{in}(\tilde{T}) \;\big|\; \mathsf{out}(\tilde{T}) \;\big|\; \forall\tilde{i}.\mathsf{serv}^K(\tilde{T}) \;\big|\; \forall\tilde{i}.\mathsf{iserv}^K(\tilde{T}) \;\big|\; \forall\tilde{i}.\mathsf{oserv}^K(\tilde{T})$$

The three different types for channels and servers correspond to the three different sets of capabilities. We note $\mathsf{serv}$ when the server have both capabilities, $\mathsf{iserv}$ when it has only input and $\mathsf{oserv}$ when it has only output. Then, for servers, we have additional information: there is a quantification over index variables, and the index $K$ stands for the *complexity* of the process spawned by this server. A typical example could be a server taking as input a list and a channel, and sending to this channel the sorted list, in time $k \cdot n$ where $n$ is the size of the list : $P = \;!a(x,b).\cdots\overline{b}\langle e\rangle$ where $e$ represents at the end of the process the list $x$ sorted. Such a server name $a$ could be given the type $\forall i.\mathsf{serv}^{k \cdot i}(\mathsf{List}[0,i](\mathcal{B}), \mathsf{out}(\mathsf{List}[0,i](\mathcal{B})))$. This type means that for all integers $i$, if given a list of size at most $i$ and an output channel waiting for a list of size at most $i$, the process spawned by this server will stop at time at most $k \cdot i$. Those bounded index variables $\tilde{i}$ are very useful especially for replicated input. As a replicated input is made to be used several times with different values, it is useful to allow this kind of polymorphism on indices. Moreover, if a replicated input is used to encode a recursion, with this polymorphism we can take into account the different recursive calls with different values and different complexities.

$$\frac{(\phi,\tilde{i});\Phi \vdash \tilde{T} \sqsubseteq \tilde{U} \qquad (\phi,\tilde{i});\Phi \vdash \tilde{U} \sqsubseteq \tilde{T} \qquad (\phi,\tilde{i});\Phi \vDash K = K'}{\phi;\Phi \vdash \forall\tilde{i}.\mathsf{serv}^K(\tilde{T}) \sqsubseteq \forall\tilde{i}.\mathsf{serv}^{K'}(\tilde{U})}$$

$$\frac{}{\phi;\Phi \vdash \forall\tilde{i}.\mathsf{serv}^K(\tilde{T}) \sqsubseteq \forall\tilde{i}.\mathsf{iserv}^K(\tilde{T})} \qquad \frac{}{\phi;\Phi \vdash \forall\tilde{i}.\mathsf{serv}^K(\tilde{T}) \sqsubseteq \forall\tilde{i}.\mathsf{oserv}^K(\tilde{T})}$$

$$\frac{(\phi,\tilde{i});\Phi \vdash \tilde{T} \sqsubseteq \tilde{U} \quad (\phi,\tilde{i});\Phi \vDash K' \leq K}{\phi;\Phi \vdash \forall\tilde{i}.\mathsf{iserv}^K(\tilde{T}) \sqsubseteq \forall\tilde{i}.\mathsf{iserv}^{K'}(\tilde{U})} \qquad \frac{(\phi,\tilde{i});\Phi \vdash \tilde{U} \sqsubseteq \tilde{T} \quad (\phi,\tilde{i});\Phi \vDash K \leq K'}{\phi;\Phi \vdash \forall\tilde{i}.\mathsf{oserv}^K(\tilde{T}) \sqsubseteq \forall\tilde{i}.\mathsf{oserv}^{K'}(\tilde{U})}$$

$$\frac{\phi;\Phi \vdash T \sqsubseteq T' \qquad \phi;\Phi \vdash T' \sqsubseteq T''}{\phi;\Phi \vdash T \sqsubseteq T''}$$

**Fig. 6.** Subtyping Rules for Server Types

Then, we describe subtyping for servers in Figure 6. As explained previously, capabilities modify the variance of types, and a channel can lose capabilities by subtyping. Subtyping for channel types can be deduced from the rules for servers. Note that the transitivity rule is not necessary and the subtyping relation could be exhaustively described. However, in order to reduce the number of rules, we present subtyping with a transitivity rule. Finally, subtyping can be extended to contexts, and we write $\Gamma \sqsubseteq \Delta$ when $\Gamma$ and $\Delta$ have the same domain and for each variable $v : T \in \Gamma$ and $v : T' \in \Delta$, we have $T \sqsubseteq T'$.

$$\frac{v : T \in \Gamma}{\phi; \Phi; \Gamma \vdash v : T} \qquad \frac{}{\phi; \Phi; \Gamma \vdash 0 : \mathsf{Nat}[0,0]} \qquad \frac{}{\phi; \Phi; \Gamma \vdash [] : \mathsf{List}[0,0](\mathcal{B})}$$

$$\frac{\phi; \Phi; \Gamma \vdash e : \mathsf{Nat}[I,J]}{\phi; \Phi; \Gamma \vdash \mathsf{s}(e) : \mathsf{Nat}[I+1,J+1]}$$

$$\frac{\phi; \Phi; \Gamma \vdash e : \mathcal{B} \qquad \phi; \Phi; \Gamma \vdash e' : \mathsf{List}[I,J](\mathcal{B})}{\phi; \Phi; \Gamma \vdash e :: e' : \mathsf{List}[I+1,J+1](\mathcal{B})}$$

$$\frac{\phi; \Phi; \Delta \vdash e : U \qquad \phi; \Phi \vdash \Gamma \sqsubseteq \Delta \qquad \phi; \Phi \vdash U \sqsubseteq T}{\phi; \Phi; \Gamma \vdash e : T}$$

**Fig. 7.** Typing Rules for Expressions

We can now present the type system. Rules for expressions are given in Figure 7. The typing for expressions $\phi; \Phi; \Gamma \vdash e : T$ means that under the constraints $\Phi$, in the context $\Gamma$, the expression $e$ can be given the type $T$. We use the notation $\phi; \Phi; \Gamma \vdash \tilde{e} : \tilde{T}$ for a sequence of typing judgements for expressions in the tuple $\tilde{e}$.

Then, rules for processes are described in Figure 8 and Figure 9. Figure 9 describes rules specific to work, whereas rules in Figure 8 will be reused for span. A typing judgement $\phi; \Phi; \Gamma \vdash P \triangleleft K$ intuitively means that under the constraints $\Phi$, in a context $\Gamma$, a process $P$ is typable and its work complexity is bounded by $K$.

The rules can be seen as a combination of input/output typing rules with rules found in a size type system. The main differences are that because of the two kinds of channels, we need two rules for an output. And, for servers, quantification over index variables should be taken in account. Note that a replicated input has complexity zero, and it is a call to this server that generates complexity in the type system. This is because once defined, a replicated input stays during all the reduction, so we do not want them to generate complexity. Note also that the pattern matching rules are the only ones which add constraints in the hypothesis, which provide information on the size in the typing. This is particularly useful for recursion. Finally, there is an explicit rule for subtyping, and in this rule we can arbitrarily increase the index corresponding to the complexity.

$$\frac{}{\phi;\Phi;\Gamma \vdash 0 \lhd 0} \qquad \frac{\phi;\Phi;\Gamma, a : T \vdash P \lhd K}{\phi;\Phi;\Gamma \vdash (\nu a)P \lhd K}$$

$$\frac{\phi;\Phi;\Gamma \vdash e : \mathsf{Nat}[I,J] \qquad \begin{array}{c}\phi;(\Phi, I \leq 0);\Gamma \vdash P \lhd K \\ \phi;(\Phi, J \geq 1);\Gamma, x : \mathsf{Nat}[I{-}1, J{-}1] \vdash Q \lhd K\end{array}}{\phi;\Phi;\Gamma \vdash \mathtt{match}(e)\ \{\mathtt{0} \mapsto P;;\ \mathtt{s}(x) \mapsto Q\} \lhd K}$$

$$\frac{\phi;\Phi;\Gamma \vdash e : \mathsf{List}[I,J](\mathcal{B}) \qquad \begin{array}{c}\phi;(\Phi, I \leq 0);\Gamma \vdash P \lhd K \\ \phi;(\Phi, J \geq 1);\Gamma, x : \mathcal{B}, y : \mathsf{List}[I{-}1, J{-}1](\mathcal{B}) \vdash Q \lhd K\end{array}}{\phi;\Phi;\Gamma \vdash \mathtt{match}(e)\ \{[] \mapsto P;;\ x :: y \mapsto Q\} \lhd K}$$

$$\frac{\phi;\Phi;\Delta \vdash P \lhd K \qquad \phi;\Phi \vdash \Gamma \sqsubseteq \Delta \qquad \phi;\Phi \vDash K \leq K'}{\phi;\Phi;\Gamma \vdash P \lhd K'}$$

**Fig. 8.** Common Typing Rules for Processes

$$\frac{\phi;\Phi;\Gamma \vdash P \lhd K \qquad \phi;\Phi;\Gamma \vdash Q \lhd K'}{\phi;\Phi;\Gamma \vdash P \mid Q \lhd K + K'} \qquad \frac{\phi;\Phi;\Gamma \vdash P \lhd K}{\phi;\Phi;\Gamma \vdash \mathtt{tick}.P \lhd K + 1}$$

$$\frac{\phi;\Phi;\Gamma \vdash a : \forall\tilde{i}.\mathsf{iserv}^K(\tilde{T}) \qquad (\phi,\tilde{i});\Phi;\Gamma,\tilde{v} : \tilde{T} \vdash P \lhd K}{\phi;\Phi;\Gamma \vdash !a(\tilde{v}).P \lhd 0}$$

$$\frac{\phi;\Phi;\Gamma \vdash a : \mathsf{in}(\tilde{T}) \qquad \phi;\Phi;\Gamma,\tilde{v} : \tilde{T} \vdash P \lhd K}{\phi;\Phi;\Gamma \vdash a(\tilde{v}).P \lhd K}$$

$$\frac{\phi;\Phi;\Gamma \vdash a : \mathsf{out}(\tilde{T}) \qquad \phi;\Phi;\Gamma \vdash \tilde{e} : \tilde{T}}{\phi;\Phi;\Gamma \vdash \overline{a}\langle\tilde{e}\rangle \lhd 0}$$

$$\frac{\phi;\Phi;\Gamma \vdash a : \forall\tilde{i}.\mathsf{oserv}^K(\tilde{T}) \qquad \phi;\Phi;\Gamma \vdash \tilde{e} : \tilde{T}\{\tilde{J}/\tilde{i}\}}{\phi;\Phi;\Gamma \vdash \overline{a}\langle\tilde{e}\rangle \lhd K\{\tilde{J}/\tilde{i}\}}$$

**Fig. 9.** Work Typing Rules for Processes

#### 3.2 Subject Reduction

We now state the properties of this typing system. We do not detail the proofs as we will be more precise in the following sections with the type system for span. In the type system for work, we can easily obtain some properties such as weakening and strengthening and that index variables can be substituted by any index in a typing derivation. Finally, we have that substitution in processes preserves typing. With those properties, we obtain the usual subject reduction.

**Theorem 1 (Subject Reduction).** *If $\phi;\Phi;\Gamma \vdash P \lhd K$ and $P \to Q$ then $\phi;\Phi;\Gamma \vdash Q \lhd K$.*

Then, we also obtain the following theorem.

**Theorem 2 (Quantitative Subject Reduction).** *If $P \to_1 Q$ and $\phi;\Phi;\Gamma \vdash P \lhd K$ then we have $\phi;\Phi;\Gamma \vdash Q \lhd K'$ with $\phi;\Phi \vDash K' + 1 \leq K$.*

So, as a consequence we almost immediately obtain that $K$ is indeed a bound on the work of $P$ if we have $\phi; \Phi; \Gamma \vdash P \triangleleft K$.

Note that this soundness result is easily adaptable to similar type systems for work. As stated before, we can enrich the type system with other algebraic data-types and the proof can easily be adapted. Moreover, we can get rid of the distinction between channels and servers and take a similar typing for both, and we still get the soundness. We decided here to present this version as an introduction for the type system for span, but the work in itself can be of interest.

For example, an interesting consequence of this soundness theorem is that it immediately gives soundness for any subsystem. In particular, we detail in the technical report [5] a (slightly) weaker typing system where the shape of types are restricted in order to have an inference procedure close to the one in [4].

## 4 Types for Parallel Complexity

We present here a type system for span, so we want as previously a type system such that typing a process gives us a bound on its span. Formally, we will prove the following theorem:

**Theorem 3 (Typing and Complexity).** *Let $P$ be a process and $m$ be its global parallel complexity. If we have $\phi; \Phi; \Gamma \vdash P \triangleleft K$, then $\phi; \Phi \vDash K \geq m$.*

Remark that this theorem talks about open processes. However, our notion of complexity does not behave well with open processes. For example the process $\mathtt{match}(v)\ \{\mathtt{0} \mapsto P;;\ \mathtt{s}(x) \mapsto Q\}$ is in normal form for a variable $v$, so this process has global complexity 0. Still, we will also obtain the following corollary:

**Corollary 1 (Complexity and Open Processes).**

- *If $\phi; \Phi; \Gamma, \tilde{v} : \tilde{T} \vdash P \triangleleft K$, then for any sequence of expressions $\tilde{e}$ such that $\phi; \Phi; \Gamma \vdash \tilde{e} : \tilde{T}$, $K$ is a bound on the global complexity of $P[\tilde{v} := \tilde{e}]$*
- *If $\phi; \Phi; \Gamma \vdash P \triangleleft K$, then for any other annotated process $Q$ such that $\phi; \Phi; \Gamma \vdash Q \triangleleft K'$, $max(K, K')$ is a bound on the global complexity of $P \mid Q$.*

So, when we give a typing $\phi; \Phi; \Gamma \vdash P \triangleleft K$ for an open process, we should not see $K$ as a bound on the actual complexity on $P$, but we should see it as a bound on the complexity of this particular process in an environment respecting the type of $\Gamma$. So, in $\phi; \Phi; v : \mathsf{Nat}[2, 10] \vdash \mathtt{match}(v)\ \{\mathtt{0} \mapsto P;;\ \mathtt{s}(x) \mapsto Q\} \triangleleft K$, $K$ is a bound on the complexity of this pattern matching under the assumption that the environment gives to $v$ an integer value between 2 and 10.

### 4.1 Size Types with Time

The type system is an extension of the previous one. In order to take into account parallelism, we need a way to synchronize the time between processes in parallel, thus we will add some time information in types, as in [27] or [9].

**Definition 8.** *The set of types and* base types *are given by the grammar:*

$$\mathcal{B} := \mathsf{Nat}[I,J] \mid \mathsf{List}[I,J](\mathcal{B})$$

$$T := \mathcal{B} \mid \mathsf{ch}_I(\tilde{T}) \mid \mathsf{in}_I(\tilde{T}) \mid \mathsf{out}_I(\tilde{T}) \mid \forall_I \tilde{i}.\mathsf{serv}^K(\tilde{T}) \mid \forall_I \tilde{i}.\mathsf{iserv}^K(\tilde{T}) \mid \forall_I \tilde{i}.\mathsf{oserv}^K(\tilde{T})$$

As before, we have channel types, server types, and input/output capabilities in those types. For a channel type or a server type, the index $I$ is called the *time* of this type. Giving a channel name the type $\mathsf{ch}_I(\tilde{T})$ ensures that communication on this channel should happen within time $I$. For example, a channel name of type $\mathsf{ch}_0(\tilde{T})$ should be used to communicate before any tick occurs. With this information, we can know when the continuation of an input will be available. Likewise, a server name of type $\forall_I \tilde{i}.\mathsf{iserv}^K(\tilde{T})$ should be used in a replicated input, and this replicated input should be ready to receive for any time greater than $I$. Typically, a process $\texttt{tick}.!a(\tilde{v}).P$ enforces that the type of $a$ is $\forall_I \tilde{i}.\mathsf{iserv}^K(\tilde{T})$ with $I$ greater than one, as the replicated input is not ready to receive at time zero.

As before, we define a notion of subtyping on those types. The rules are essentially the same as the ones in Figures 5 and 6. The only difference is that we force the time of a type to be invariant in subtyping.

In order to write the typing rules, we need some other definitions to work with time in types. The first thing we need is a way to advance time.

**Definition 9 (Advancing Time in Types).** *Given a set of index variables $\phi$, a set of constraints $\Phi$, a type $T$ and an index $I$, we define $T$ after $I$ time units, denoted $\langle T\rangle_{-I}^{\phi;\Phi}$ by:*

- $\langle \mathcal{B}\rangle_{-I}^{\phi;\Phi} = \mathcal{B}$
- $\langle \mathsf{ch}_J(\tilde{T})\rangle_{-I}^{\phi;\Phi} = \mathsf{ch}_{(J-I)}(\tilde{T})$ *if $\phi;\Phi \vDash J \geq I$. It is undefined otherwise. Other channel types follow exactly the same pattern.*
- $\langle \forall_J \tilde{i}.\mathsf{serv}^K(\tilde{T})\rangle_{-I}^{\phi;\Phi} = \forall_{(J-I)} \tilde{i}.\mathsf{serv}^K(\tilde{T})$ *if $\phi;\Phi \vDash J \geq I$. Otherwise, $\langle \forall_J \tilde{i}.\mathsf{serv}^K(\tilde{T})\rangle_{-I}^{\phi;\Phi} = \forall_{(J-I)} \tilde{i}.\mathsf{oserv}^K(\tilde{T})$*
- $\langle \forall_J \tilde{i}.\mathsf{iserv}^K(\tilde{T})\rangle_{-I}^{\phi;\Phi} = \forall_{(J-I)} \tilde{i}.\mathsf{iserv}^K(\tilde{T})$ *if $\phi;\Phi \vDash J \geq I$. It is undefined otherwise.*
- $\langle \forall_J \tilde{i}.\mathsf{oserv}^K(\tilde{T})\rangle_{-I}^{\phi;\Phi} = \forall_{(J-I)} \tilde{i}.\mathsf{oserv}^K(\tilde{T})$.

*This definition can be extended to contexts, with $\langle v : T, \Gamma\rangle_{-I}^{\phi;\Phi} = v : \langle T\rangle_{-I}^{\phi;\Phi}, \langle \Gamma\rangle_{-I}^{\phi;\Phi}$ if $\langle T\rangle_{-I}^{\phi;\Phi}$ is defined. Otherwise, $\langle v : T, \Gamma\rangle_{-I} = \langle \Gamma\rangle_{-I}^{\phi;\Phi}$. We will often omit the $\phi;\Phi$ in the notation when it is clear from the context.*

*Recall that as the order $\leq$ on indexes is not total, $\phi;\Phi \nvDash J \geq I$ does not mean that $\phi;\Phi \vDash J < I$.*

Let us explain a bit the definition here. For base types, there is no time indication thus nothing happens. Then, one can wonder what happens when the time of $T$ is not greater than $I$. For non-server channel types, we consider that their time is over, thus we erase them from the context. For servers this is a bit more complicated. Indeed, when a server is defined, it must stay available until the end. Thus, an output to a server should always be possible, no matter the time. Still, the input capability of a server should not be available eternally, as the time $I$ is supposed to mean the time for which a replicated input is effectively defined. So, when this time has passed, we should not be able to define a replicated input any more.

**Definition 10 (Time Invariant Context).** *Given a set of index variables* $\phi$ *and a set of constraints* $\Phi$*, a context* $\Gamma$ *is said to be* time invariant *when it only contains base type variables or output server types* $\forall_I \tilde{i}.\mathsf{oserv}^K(\tilde{T})$ *with* $\phi;\Phi \vDash I = 0$.

Such a context is thus invariant by the operator $\langle\cdot\rangle_{-I}$ for any $I$. This is typically the kind of context that we need to define a server, as a server should not be dependent on the time it is called. We can now present the type system. Typing rules for expressions and some processes do not change, they can be found in Figure 7 and Figure 8. In Figure 10, we present the remaining rules in this type system that differ from the ones in Figure 9. As before, a typing judgement $\phi;\Phi;\Gamma \vdash P \triangleleft K$ intuitively means that under the constraints $\Phi$, in a context $\Gamma$, a process $P$ is typable and its span complexity is bounded by $K$.

$$\frac{\phi;\Phi;\Gamma \vdash P \triangleleft K \qquad \phi;\Phi;\Gamma \vdash Q \triangleleft K}{\phi;\Phi;\Gamma \vdash P \mid Q \triangleleft K} \qquad \frac{\phi;\Phi;\langle\Gamma\rangle_{-1} \vdash P \triangleleft K}{\phi;\Phi;\Gamma \vdash \mathtt{tick}.P \triangleleft K+1}$$

$$\frac{\phi;\Phi;\Gamma,\Delta \vdash a : \forall_I \tilde{i}.\mathsf{iserv}^K(\tilde{T}) \qquad \begin{array}{c}\phi;\Phi \vdash \langle\Gamma\rangle_{-I}^{\phi;\Phi} \sqsubseteq \Gamma' \text{ and } \Gamma' \text{ time invariant} \\ \phi,\tilde{i};\Phi;\Gamma',\tilde{v} : \tilde{T} \vdash P \triangleleft K\end{array}}{\phi;\Phi;\Gamma,\Delta \vdash !a(\tilde{v}).P \triangleleft I}$$

$$\frac{\phi;\Phi;\Gamma \vdash a : \mathsf{in}_I(\tilde{T}) \qquad \phi;\Phi;\langle\Gamma\rangle_{-I},\tilde{v} : \tilde{T} \vdash P \triangleleft K}{\phi;\Phi;\Gamma \vdash a(\tilde{v}).P \triangleleft K + I}$$

$$\frac{\phi;\Phi;\Gamma \vdash a : \mathsf{out}_I(\tilde{T}) \qquad \phi;\Phi;\langle\Gamma\rangle_{-I} \vdash \tilde{e} : \tilde{T}}{\phi;\Phi;\Gamma \vdash \overline{a}\langle\tilde{e}\rangle \triangleleft I}$$

$$\frac{\phi;\Phi;\Gamma \vdash a : \forall_I \tilde{i}.\mathsf{oserv}^K(\tilde{T}) \qquad \phi;\Phi;\langle\Gamma\rangle_{-I} \vdash \tilde{e} : \tilde{T}\{\tilde{J}/\tilde{i}\}}{\phi;\Phi;\Gamma \vdash \overline{a}\langle\tilde{e}\rangle \triangleleft K\{\tilde{J}/\tilde{i}\} + I}$$

**Fig. 10.** Span Typing Rules for Processes

The rule for parallel composition shows that we consider parallel complexity as we take the maximum between the two processes instead of the sum. In practice, we ask for the same complexity $K$ in both branches of parallel composition, but with the subtyping rule, it corresponds indeed to the maximum. For input server, we integrate some weakening on context ($\Delta$), and we want a time invariant context to type the server, as a server should not depend on time. Weakening is important since some types are not time invariant, such as channels. So, we need to separate time invariant types that can be used in the continuation $P$ from other types.

Some rules make the time advance in their continuation, for example the tick rule or input rule. This is expressed by the advance time operator on contexts, and because time advances, the complexity also increases. Also, remark that because of the advance of time, some channels name could disappear, thus there is a kind of "time uniqueness" for channels, contrary to the previous section. This will be detailed later. Also, note that in the rule for replicated input, there is an explicit subtyping in the premises. This is because $\langle \Gamma \rangle_{-I}^{\phi;\Phi}$ is not time invariant, since the type of $a$ is at least $\forall_0 \tilde{i}.\mathsf{iserv}^K(\tilde{T})$ in this case. However, if this server had both input and output capabilities, we can give a time invariant type for $a$ (or other servers) just by removing the input capability, which can be done by subtyping.

Looking back at Corollary 1, we can for example understand the rule for input by taking the judgement $\phi; \Phi; a : \mathsf{ch}_3() \vdash a().\mathtt{tick}.0 \lhd 4$. This expresses that with an environment providing a message on $a$ within 3 times units, this process terminates in 4 time units.

Finally, we can see that if we remove all size annotations and merge server types and channel types together, we get back the classical input/output types, and all the rules described here are admissible in the classical input/output type system for the $\pi$-calculus.

#### 4.2 Examples

**An Example to Justify the Use of Time.** In order to justify the use of time in types for span, and to show how we could find the time of a channel, we present here three examples of recursive calls with different behaviour. We do not detail here a typing derivation, a more detailed example will be described later, in Section 5. Usually, type inference for a size system reduces to satisfying a set of constraints on indices. We believe that even with time indexes on channels, type inference is still reducible to satisfying such a set of constraints. So, for the sake of simplicity, we will describe this example with constraints.

We define three processes $P_1, P_2$ and $P_3$ by:

$$P_l \equiv !a(n,r).\mathtt{tick}.\mathtt{match}(n)\ \{\mathtt{0} \mapsto \overline{r}\langle\rangle;;\ \mathtt{s}(m) \mapsto (\nu r')(\nu r'')(Q_l)\}$$

for the following definition of $Q_i$:

$$Q_1 \equiv \overline{a}\langle m, r'\rangle \mid \overline{a}\langle m, r''\rangle \mid r'().r''().\overline{r}\langle\rangle$$
$$Q_2 \equiv \overline{a}\langle m, r'\rangle \mid r'().\overline{a}\langle m, r''\rangle \mid r''().\overline{r}\langle\rangle$$
$$Q_3 \equiv \overline{a}\langle m, r'\rangle \mid r'().(\overline{a}\langle m, r''\rangle \mid \overline{r}\langle\rangle) \mid r''().0$$

So intuitively, for $P_1$ the two recursive calls are done after one unit of time in parallel, and the return signal on $r$ is done when both processes have done their return signal on $r'$ and $r''$. So, this is total parallelism for the two recursive calls (the span is linear in $n$). For $P_2$, a first recursive call is done, and then the process waits for the return signal on $r'$, and when it receives it, the second recursive call begins. So, this is totally sequential (the span is exponential in $n$). Finally, for $P_3$ we have an intermediate situation between totally parallel and totally sequential. The process starts with a recursive call. Then, it waits for the return signal on $r'$. When this signal arrives, it immediately starts the second recursive call and immediately does the return signal on $r$. So, intuitively, the second recursive call starts when all the "left" calls have been done. Note that those three servers have the same work, which is exponential in $n$.

So, let us type the three examples with the type system for span. For the sake of simplicity, we omit the typing of expressions, we only consider the difficult branch for the match constructors, and we focus on complexity and time. We consider the following context that is used for the three processes:

$$\Gamma \equiv a : \forall_0 i.\mathsf{oserv}^{f(i)}(\mathsf{Nat}[0,i], \mathsf{ch}_{g(i)}()), n : \mathsf{Nat}[0,i], r : \mathsf{ch}_{g(i)}$$

We have two unknown function symbols: $f$, that represents the complexity of the server, and $g$, the time for the return channel. We also use this second context:

$$\Delta \equiv \langle \Gamma \rangle_{-1}, m : \mathsf{Nat}[0, i{-}1], r' : \mathsf{ch}_{g'(i)}, r'' : \mathsf{ch}_{g''(i)}$$

This gives two more unknown functions, $g'$ and $g''$ corresponding respectively to the time of $r'$ and $r''$ when defined. The three processes start with the same typing. We use a double line to express that we do not use a real typing rule, so we can omit some premises or do simultaneously several typing rules.

$$\dfrac{\dfrac{\dfrac{i; i \geq 1; \Delta \vdash Q_l \triangleleft f(i){-}1}{i; \cdot; \langle \Gamma \rangle_{-1} \vdash \mathtt{match}(n)\ \{0 \mapsto \overline{r}\langle\rangle;;\ \mathtt{s}(m) \mapsto (\nu r')(\nu r'')(Q_i)\} \triangleleft f(i){-}1}}{i; \cdot; \Gamma \vdash \mathtt{tick.match}(n)\ \{0 \mapsto \overline{r}\langle\rangle;;\ \mathtt{s}(m) \mapsto (\nu r')(\nu r'')(Q_l)\} \triangleleft f(i)}}{\cdot; \cdot; a : \forall_0 i.\mathsf{oserv}^{f(i)}(\mathsf{Nat}[0,i], \mathsf{ch}_{g(i)}()) \vdash P_l \triangleleft 0}$$

The first thing to remark is that the typing does a `tick` typing rule. In this rule for `tick`, the complexity on the bottom should have the shape $K + 1$ for some $K$, so here we obtain immediately that $f(i) \geq 1$. In the same way, $r$ should still be defined in $\langle \Gamma \rangle_{-1}$, so by definition of time advance, it means that $g(i) \geq 1$.

Then, for the three processes, the typing gives the following conditions on the indices, for $i \geq 1$. For $Q_1$:

$$f(i){-}1 \geq f(i{-}1) \qquad g'(i) = g(i{-}1) \qquad g''(i) = g(i{-}1)$$

$$g''(i) \geq g'(i) \qquad g(i){-}1 \geq g''(i) \qquad f(i){-}1 \geq g(i){-}1$$

The first constraint is because the total complexity $f(i){-}1$ must be greater than the complexity of the two recursive calls $f(i{-}1)$. Then, $r'$ and $r''$ must have a time equal to $g(i{-}1)$ in order to correspond to the type of $a$ in the outputs $\overline{a}\langle m, r'\rangle$ and $\overline{a}\langle m, r''\rangle$. Finally, as $r''$ waits for input after $r'$, the time of $r''$ must be greater than the time of $r'$. Similarly, the time of $r$ (which is equal to $g(i){-}1$) must be greater than the time of $r''$, and the total complexity $f[i]{-}1$ must be greater than the complexity of $r'().r''().\overline{r}\langle\rangle$ which is equal to the time of $r$. So, we can satisfy the conditions with the following choice:

$$f(i) \equiv i+1 \qquad g(i) \equiv i+1 \qquad g'(i) \equiv g''(i) \equiv i$$

So, as expected, the span, represented by the function $f$, is indeed linear.

Then, for $Q_2$, the second call is delayed of $g'(i)$ time units because we need to wait for $r'$. Thus, we obtain the following constraints.

$$f(i)\text{-}1 \geq f(i\text{-}1) \qquad g'(i) = g(i\text{-}1) \qquad f(i)\text{-}1 \geq g'(i) + f(i\text{-}1)$$
$$g''(i)\text{-}g'(i) = g(i\text{-}1) \qquad g(i)\text{-}1 \geq g''(i) \qquad f(i)\text{-}1 \geq g(i)\text{-}1$$

This delay of $g'(i)$ time units can be seen in the third and fourth constraints. Again, the third constraint is because the complexity should be greater that the complexity of the second call, and the type of $r''$ should correspond to the type in $a$. Thus, we can take

$$f(i) \equiv 2^{i+1}\text{-}1 \qquad g(i) \equiv 2^{i+1}\text{-}1$$

So, we indeed obtain the exponential complexity.

However, with those two examples, the time of the channel $r$ is always equal to the complexity of the server $a$, so we cannot really see the usefulness of time. Still, with the next example we obtain something more interesting. So, for $Q_3$, this time the fifth constraint on $g(i)$ (depending on when the output to $r$ is done) is different, and we obtain:

$$f(i)\text{-}1 \geq f(i\text{-}1) \qquad g'(i) = g(i\text{-}1) \qquad f(i)\text{-}1 \geq g'(i) + f(i\text{-}1)$$
$$g''(i)\text{-}g'(i) = g(i\text{-}1) \qquad g(i)\text{-}1 \geq g'(i) \qquad f(i)\text{-}1 \geq g(i)\text{-}1 \qquad f(i)\text{-}1 \geq g''(i)$$

The last constraint is because, again, the complexity should be greater that the complexity of calling $r''$. So, using the equalities, and by removing redundant inequalities, we obtain for $f$ and $g$:

$$f(i) \geq 1 + g(i\text{-}1) + f(i\text{-}1) \qquad g(i) \geq 1 + g(i\text{-}1) \qquad f(i) \geq 1 + 2 \cdot g(i\text{-}1)$$

Thus, we can take:

$$g(i) \equiv i+1 \qquad f(i) \equiv \frac{(i+1)(i+2)}{2}$$

The complexity is quadratic in $n$. Note that for this example, the complexity $f$ depends directly on $g$, and $g$ is given by a recursive equation independent of $f$. So in a sense, to find the complexity, we need to find first the delay of the second recursive call. Without time indications on channel, it would not be possible to track and obtain this recurrence relation on $g$ and thus we could not deduce the complexity.

Note that the two first examples used channels as a return signal for a parallel computation, whereas for the last example, channels are used as a synchronization point in the middle of a computation. We believe that this flexibility of channels justifies the use of $\pi$-calculus to reason about parallel computation. Moreover, this work is a step to a more expressive type system inspired by [27], taking in account concurrent behaviour. Indeed, as we will show, the current type system fails to capture some simple concurrency.

**Limitations of the Type System.** Our current type system enforces some kind of time uniqueness in channels. Indeed, take the process $a().\texttt{tick}.\overline{a}\langle\rangle$. When trying to type this process, we obtain:

$$\dfrac{\dfrac{\cdot;\cdot \vdash \mathsf{ch}_I() \sqsubseteq \mathsf{in}_I()}{\cdot;\cdot;a:\mathsf{ch}_I() \vdash a:\mathsf{in}_I()} \qquad \dfrac{\dfrac{\text{Error}}{\cdot;\cdot;\langle a:\mathsf{ch}_0()\rangle_{-1} \vdash \overline{a}\langle\rangle \triangleleft 0}}{\cdot;\cdot;a:\mathsf{ch}_0() \vdash \texttt{tick}.\overline{a}\langle\rangle \triangleleft 1}}{\cdot;\cdot;a:\mathsf{ch}_I() \vdash a().\texttt{tick}.\overline{a}\langle\rangle \triangleleft I+1}$$

As by definition $\langle a:\mathsf{ch}_0()\rangle_{-1}$ is $\emptyset$, we cannot type the output on $a$. So, channels have strong constraints on the time they can be used. This is true especially when channels are not used linearly. Still, note that we can type a process of the shape $a().0 \mid \overline{a}\langle\rangle \mid \texttt{tick}.\overline{a}\langle\rangle$, so it is better than plain linearity on channels. This restriction limits examples of concurrent behaviours. For example, take two processes $P_1$ and $P_2$ that should be executed but not simultaneously. In order to do that in a concurrent setting, we can use semaphores. In $\pi$-calculus, we could consider the process $(\nu a)(a().P_1' \mid a().P_2' \mid \overline{a}\langle\rangle)$, where $P_1'$ is $P_1$ with an output $\overline{a}\langle\rangle$ at the end, likewise for $P_2'$. This is a way to simulate semaphore in $\pi$-calculus. Now, we can see that this example has the same problem as the example given above if for example $P_1$ contains a `tick`, thus we cannot type this kind of processes.

Still, we believe that for parallel computation, our type system should be quite expressive in practice. Indeed, as stated above, the restriction appears especially when channels are not used linearly. However, it is known that linear $\pi$-calculus in itself is expressive for parallel computation [31]. For example, classical encodings of functional programs in a parallel setting rely on the use of linear return signals, as we will see in the example for bitonic sort in Sect. 5. Moreover, session types can also be encoded in linear $\pi$-calculus in the presence of variant types [28,8]. Note that in order to encode a calculus as the one in [9], we would also need recursive types. Our calculus and its proof of soundness could be extended to variant types, but not straightforwardly to recursive types. However, we believe the results on the linear $\pi$-calculus we cited suggest that the restriction given above should not be too harmful for parallel computation.

#### 4.3 Complexity Results

In this section, we show how to prove that our type system indeed gives a bound on the number of time reduction steps of a process following the maximal progress assumption. We only give in this section intuitions about the proofs. The detailed proofs can be found in the technical report [5].

In the following section, as we will work with the reduction $\Rightarrow_p$, we need to consider annotated processes instead of simple processes. So, we need to enrich our type system with a rule for the constructor $n : P$.

$$\frac{\phi;\Phi;\langle\Gamma\rangle_{-n} \vdash P \triangleleft K}{\phi;\Phi;\Gamma \vdash n : P \triangleleft K+n}$$

As the intuition suggested, this rule is equivalent to $n$ times the typing rule for `tick`. We can now work on the properties of our type system on annotated processes.

The procedure to prove the subject reduction for $\Rightarrow_p$ in this type system is intrinsically more difficult than the one for Theorem 1. So, from the proof of subject reduction for span, one could deduce the proof of subject reduction for work, just by forgetting the consideration with time and the constructor $n : P$ in the following proof. Thus, in the technical report, only the proof for span is detailed.

Again, we have both weakening and strengthening in this type system. We also have a property specific to size type systems, expressing that an index variable can be substituted by any index. We also need a lemma specific to the notion of time.

**Definition 11 (Delaying).** *Given a type $T$ and an index $I$, we define the* delaying *of $T$ by $I$ units of time, denoted $T_{+I}$:*

$$\mathcal{B}_{+I} = \mathcal{B} \qquad (\mathsf{ch}_J(\tilde{T}))_{+I} = \mathsf{ch}_{J+I}(\tilde{T})$$

*and for other channel and server types, the definition is in correspondence with the one on the right above. This definition can be extended to contexts.*

**Lemma 2 (Delaying).** *If $\phi; \Phi; \Gamma \vdash P \triangleleft K$ then $\phi; \Phi; \Gamma_{+I} \vdash P \triangleleft K + I$.*

With this lemma, we can see that if we add a delay of $I$ time units in the contexts for all channels, it increases the complexity by $I$ time units, thus we see the link between time in types and the complexity. Then, we can show the usual substitution lemma.

**Lemma 3 (Substitution).**

1. *If $\phi; \Phi; \Gamma, v : T \vdash e' : U$ and $\phi; \Phi; \Gamma \vdash e : T$ then $\phi; \Phi; \Gamma \vdash e'[v := e] : U$.*
2. *If $\phi; \Phi; \Gamma, v : T \vdash P \triangleleft K$ and $\phi; \Phi; \Gamma \vdash e : T$ then $\phi; \Phi; \Gamma \vdash P[v := e] \triangleleft K$.*

Finally, we can show that typing behaves well with congruence.

**Lemma 4 (Congruence and Typing).** *Let $P$ and $Q$ be annotated processes such that $P \equiv Q$. Then, $\phi; \Phi; \Gamma \vdash P \triangleleft K$ if and only if $\phi; \Phi; \Gamma \vdash Q \triangleleft K$.*

And with all this, we obtain the subject reduction.

**Theorem 4 (Subject Reduction).** *If $\phi; \Phi; \Gamma \vdash P \triangleleft K$ and $P \Rightarrow_p Q$ then $\phi; \Phi; \Gamma \vdash Q \triangleleft K$.*

The proof is done by induction on $P \Rightarrow_p Q$. The proof can be rather tedious because of subtyping and input/output types that generate a lot of cases for subtyping, and, as expected, the most difficult cases are for communications.

Now that we have the subject reduction for $\Rightarrow_p$, we can easily deduce a more generic form of Theorem 3.

**Theorem 5.** *Let $P$ be an annotated process and let $m$ be its global parallel complexity. Then, for a typing $\phi; \Phi; \Gamma \vdash P \lhd K$, we have $\phi; \Phi \vDash K \geq m$.*

Corollary 1 is then obtained with the substitution lemma and the rule for parallel composition.

### 5 An Example: Bitonic Sort

As an example for this type system, we show how to obtain the bound on a common parallel algorithm: bitonic sort [1]. The particularity of this sorting algorithm is that it admits a parallel complexity in $\mathcal{O}(log(n)^2)$. We will show here that our type system allows to derive this bound for the algorithm, just as the paper-and-pen analysis. Actually we consider here a version for lists, which is not optimal for the number of operations, but we obtain the usual number of comparisons. For the sake of simplicity, we present here the algorithm for lists of size a power of 2. Let us briefly sketch the ideas of this algorithm. For a formal description see [1].

- A *bitonic sequence* is either a sequence composed of an increasing sequence followed by a decreasing sequence (e.g. [2, 7, 23, 19, 8, 5]), or a cyclic rotation of such a sequence (e.g. [8, 5, 2, 7, 23, 19]).
- The algorithm uses 2 main functions, `bmerge` and `bsort`.
- `bmerge` takes a bitonic sequence and recursively sorts it, as follows:
  Assume $s = [a_0, \dots, a_{n-1}]$ is a bitonic sequence such that $[a_0, \dots, a_{n/2-1}]$ is increasing and $[a_{n/2} \dots, a_{n-1}]$ is decreasing, then we consider:
  $s_1 = [\min(a_0, a_{n/2}), \min(a_1, a_{n/2+1}) \dots, \min(a_{n/2-1}, a_{n-1})]$
  $s_2 = [\max(a_0, a_{n/2}), \max(a_1, a_{n/2+1}) \dots, \max(a_{n/2-1}, a_{n-1})]$
  Then we have: $s_1$ and $s_2$ are bitonic and satisfy: $\forall x \in s_1, \forall y \in s_2, x \leq y$.
  `bmerge` then applies recursively to $s_1$ and $s_2$ to produce a sorted sequence.
- `bsort` takes a sequence and recursively sorts it. It starts by separating the sequence in two. Then, it recursively sorts the first sequence in increasing order, and the second sequence in decreasing order. With this, we obtain a bitonic sequence that can be sorted with `bmerge`.

We will encode this algorithm in $\pi$-calculus with a boolean type. As expressed before, our results can easily be extended to support boolean with a conditional constructor.

First, we suppose that a server for comparison `lessthan` is already implemented. We start with `bcompare` such that given two lists of same length, it creates the list of maximum and the list of minimum. This is described in Figure 11.

We present here intuitively the typing. To begin with, we suppose that `lessthan` is given the server type ${}_0\mathsf{oserv}^0(\mathcal{B}, \mathcal{B}, \mathsf{ch}_0(\mathsf{Bool}))$, saying that this is a server ready to be called, and it takes in input a channel that is used to return the boolean value. With this, we can give to `bcompare` the following server type:

$$\forall_0 i.\mathsf{serv}^1(\mathsf{List}[0,i](\mathcal{B}), \mathsf{List}[0,i](\mathcal{B}), \mathsf{out}_1(\mathsf{List}[0,i](\mathcal{B}), \mathsf{List}[0,i](\mathcal{B})))$$

```
!bcompare(l1,l2,a). match(l1) {
  [] ↦ ā⟨l1,l2⟩ ;;
  x :: l1' ↦ match(l2) {
    [] ↦ ā⟨l1,l2⟩ ;;
    y :: l2' ↦ (νb)(νc)(
      bcompare‾⟨l1',l2',b⟩ | tick.lessthan‾⟨x,y,c⟩
      | b(lm,lM).c(z).if z then ā⟨x :: lm,y :: lM⟩ else ā⟨y :: lm,x :: lM⟩
    )
  }
}
!bmerge(up,l,a). match(l) {
  [] ↦ ā⟨l⟩ ;;
  [y] ↦ ā⟨l⟩ ;;
  _ ↦ let (l1,l2) = partition(l) in (νb)(νc)(νd)(
    bcompare‾⟨l1,l2,b⟩ | b(p1,p2).(bmerge‾⟨up,p1,c⟩ | bmerge‾⟨up,p2,d⟩)
    | c(q1).d(q2). if up then let l' = q1 @ q2 in ā⟨l'⟩
                         else let l' = q2 @ q1 in ā⟨l'⟩
  )
}
!bsort(up,l,a). match(l) {
  [] ↦ ā⟨l⟩ ;;
  [y]↦ ā⟨l⟩ ;;
  _ ↦ let (l1,l2) = partition(l) in (νb)(νc)(νd)(
    bsort‾⟨tt,l1,b⟩ | bsort‾⟨ff,l2,c⟩
    | b(q1).c(q2).let q = q1 @ q2 in bmerge‾⟨up,q,d⟩ | d(p).ā⟨p⟩
  )
}
```

**Fig. 11.** Bitonic Sort

The important things to notice is that this server has complexity 1, and the channel taken in input has a time 1. In order to verify that this type is correct, we would first need to apply the rule for replicated input. Let us denote by $\Gamma$ the hypothesis on those two servers names, and $\Gamma'$ be as $\Gamma$ except that for `bcompare` we only have the output capability. Then, $\Gamma'$ is indeed time invariant, and we have $\vdash \langle \Gamma \rangle_{-0} \sqsubseteq \Gamma'$, so we can continue the typing with this context $\Gamma'$. Then, we need to show that the process after the replicated input indeed has complexity 1. In the cases of empty list, this can be done easily. In the non-empty case, for the $\nu$ constructor, we must give a type to the channels $b$ and $c$. We use:

$$b : \mathsf{ch}_1(\mathsf{List}[0, i{-}1](\mathcal{B}), \mathsf{List}[0, i{-}1](\mathcal{B})) \qquad c : \mathsf{ch}_1(\mathsf{Bool})$$

And we can then type the different processes in parallel.

– For the call to `bcompare`, the arguments have the expected type, and this call has complexity 1 because of the type of `bcompare`.

- For the process $\mathtt{tick}.\overline{\mathtt{lessthan}}\langle x, y, c\rangle$, the tick enforces a decreasing of time 1 in the context. This modifies in particular the time of $c$, that becomes 0. Thus, we can do the call to `lessthan` as everything is well-typed.
- Finally, for the last process, because $b$ has a time equal to 1, the first input has complexity 1 and it enforces again a decreasing of 1 time unit. In particular, the times of $c$ and $a$ become 0. Then, as there is no more `tick` and all channels have time 0, the typing proceeds without difficulties.

So, we can indeed give this server type to `bcompare`, and thus we can call this server and it generates a complexity of 1.

Then, to present the process for bitonic sort, let us use the macro $\mathtt{let}\ \tilde{v} = f(\tilde{e})\ \mathtt{in}\ P$ to represent $(\nu a)(\overline{f}\langle\tilde{e}, a\rangle \mid a(\tilde{v}).P)$, and let us also use a generalized pattern matching. We also assume that we have a function for concatenation of lists and a function `partition` taking a list of size $2n$, and giving two lists corresponding to the first $n$ elements and the last $n$ elements. Then, the process for bitonic sort is given in Figure 11.

Without going into details, the main point in the typing of those relations is to find a solution to a recurrence relation for the complexity of server types. In the typing of `bmerge`, we suppose given a list of size smaller than $2^i$ and we choose both the complexity of this type and the time of the channel $a$ equal to a certain index $K$ (with $i$ free in $K$). So, it means we chose for `bmerge` the type:

$$\forall_0 i.\mathsf{serv}^K(\mathsf{Bool}, \mathsf{List}[0, 2^i](\mathcal{B}), \mathsf{out}_K(\mathsf{List}[0, 2^i](\mathcal{B})))$$

Then, the typing gives us the following condition.

$$i \geq 1 \text{ implies } K \geq 1 + K\{i\text{-}1/i\}$$

Indeed, the two recursive calls to `bmerge` are done after one unit of time (because the input $b(p_1, p_2)$ takes one unit of time, as expressed by the type of `bcompare`), and with a list of size $2^{i-1}$. And then, the continuation after those recursive calls (the process after $c(q_1).d(q_2)$) does not generate any complexity. So, we can take $K = i$, and thus `bmerge` has logarithmic complexity. Then, in the same way we obtain a recurrence relation for the complexity $K'$ of `bsort` on an input list of size smaller than $2^i$.

$$i \geq 1 \text{ implies } K' \geq K'\{i\text{-}1/i\} + i$$

Again, the two recursive calls are done on lists of size $2^{i-1}$. This time, the delay of $i$ in the recurrence relation is given by the continuation, because of the call to `bmerge` that generates a complexity of $i$. Thus, we can take a $K'$ in $\mathcal{O}(i^2)$, and we obtain in the end that bitonic sort is indeed in $\mathcal{O}(log(n)^2)$ on a list of size $n$.

Remark that in this example, the type system gives recurrence relations corresponding to the usual recurrence relations we would obtain with a complexity analysis by hand. Here, the recurrence relation is only on $K$ because channel names are only used as return channels, so their time is always equal to the complexity of the server that uses them. In general this is not the case as we saw before, so we obtain in general mutually recurrent relations when defining a server.

### 6 Related Work

An analysis of the complexity of parallel functional programs based on types has been carried out in [23]. Their system can analyse the work and the span (called depth in this paper), and makes use of amortized complexity analysis, which allows to obtain sharp bounds. However, the kind of parallelism they analyse is limited to parallel composition. So on the one hand we are considering a more general model of parallelism, and on the other hand we are not taking advantage of amortized analysis as they do. The paper [17] proposes a complexity analysis of parallel functional programs written in interaction nets, a graph-based language derived from linear logic. Their analysis is based on size types. However, their model is also quite different from ours as interaction nets do not provide name-passing.

Other works like [2] tackle the problem of analysing the parallel complexity of a distributed system by building a distributed flow graph and searching for a path of maximal cost in this graph. Another approach to analyse loops with concurrency in an actor-based language is done by *rely-guarantee reasoning* [3]. Those approaches give interesting results on some classes of systems, but they cannot be directly applied to the $\pi$-calculus language we are considering, with dynamic creation of processes and channels. Moreover, they do not offer the same compositionality as analysis based on type systems. The paper [16] studies distributed systems that are comparable to those of [2], and analyses their complexity by means of a behaviour type system. In a second step the types are used to run an analysis that returns complexity bounds. So this approach is more compositional than that of [2], but still does not apply to our $\pi$-calculus language.

Let us now turn to related works in the setting of $\pi$-calculus or process calculi. To our knowledge, the first work to study parallel complexity in $\pi$-calculus by types was given by Kobayashi [27], as another application of his type system for deadlock freedom, further developed in other papers [30]. In his setting, channels are typed with *usages*, which are simple CCS-like processes to describe the behaviour of a channel. In order to carry out complexity analysis, those usages are annotated by two time informations, *obligation* and *capability*. The obligation level is the time at which a channel is ready to perform an action, and the capability level is the time at which it successfully finds a communication partner. We believe that when they are not infinite, the sum of those levels is related to our own time annotation of channels. The definition of parallel complexity in this work differs from ours, as it loses some non-deterministic paths and the extension with dependent types is suggested but not detailed. It is not clear to us if everything can be adapted to reason only about our parallel complexity, but we plan to study it in future work. More recently Das et al. in [9,10] proposed a type system with temporal session types to capture several parallel cost models with the use of a *tick* constructor. Our usage of time was inspired by their types with the usual *next* modality of temporal logic, but in this paper they also use the *always* and *eventually* modalities to gain expressivity. We believe that because our usage of time is more permissive, those modalities would not be useful in our calculus. Because of session-types, they have linearity for the use of data-types such as lists, but they obtain deadlock-freedom contrary to our calculus. Moreover, they provide decidable operations to simplify the use of their types, such as subtyping, but they do not define dependent types nor size types that are useful to treat data-types. Still, they provide a significant number of examples to show the expressivity of their type system.

The methodology of our work is inspired by implicit computational complexity, which aims at characterizing complexity classes by means of dedicated programming languages, mainly in the sequential setting, for instance by providing languages for FPTIME functions. Some results have been adapted to the concurrent case, but mainly for the work complexity or for other languages than the $\pi$-calculus, e.g. [32,14,7] (the latter reference is for a higher-order $\pi$-calculus). The paper [13] is closer to our setting as it defines a notion of causal complexity in $\pi$-calculus and gives a type system characterizing processes with polynomial complexity. However, contrarily to those works we do not restrict to a particular complexity class (like FPTIME) and we handle the case of the span.

Technically, the types we use are inspired from linear dependent types [6]. Those are one of the many variants of size types, which were introduced in [26].

### 7 Perspectives

We see several possible future directions to this work:

– Type inference: we plan to investigate how type inference could be automatized or partially automatized for the span type system. We will study typing by constraint generation and explore whether existing off-the-shelf solvers or new procedures could allow to solve these constraints. Preliminary results (see [5]) show that the case of work is manageable, and it generates a set of constraints close to the one in [4]. However, the case of span could require more sophisticated reasoning because of the strong distinction between servers and channels with the advancing of time.
– We have mentioned that our type system for span is not adapted to analyse some concurrent systems such as the simple example of the semaphore (Sect. 4.2). However, we believe that a type system based on an adaptation of usages [27,30,29] could be promising for this purpose.
– It would be challenging to examine whether similar type systems could be developed to account for some other complexity properties, for instance to extract the number of parallel processes needed to achieve the span.

**Acknowledgements** We are grateful to Naoki Kobayashi for suggesting the definition of annotated processes and their reduction that we use in this paper.

This work was supported by the LABEX MILYON (ANR-10-LABX-0070) of Universite de Lyon.
